# Morphometric analysis and bioclimatic distribution of *Glebionis coronaria* s.l. (Asteraceae) in the Mediterranean area

**DOI:** 10.3897/phytokeys.81.11995

**Published:** 2017-06-26

**Authors:** Eusebio Cano, Carmelo Maria Musarella, Ana Cano-Ortiz, José Carlos Piñar Fuentes, Giovanni Spampinato, Carlos José Pinto Gomes

**Affiliations:** 1 Dpt. of Animal and Plant Biology and Ecology, Section of Botany, University of Jaén, Campus Universitario Las Lagunillas s/n, 23071 Jaén, Spain; 2 Dpt. of AGRARIA, “Mediterranea” University of Reggio Calabria, Località Feo di Vito, 89122 Reggio Calabria, Italy; 3 Dpt. of Landscape, Environment and Planning; Institute for Mediterranean Agrarian and Environmental Sciences (ICAAM); School of Science and Technology, University of Évora (Portugal). Rua Romão Ramalho, n°59, P-7000-671 Évora, Portugal

**Keywords:** Bioclimatic Distribution, Biogeography, *Glebionis*, Identification Key, Micromorphology, Nomenclature

## Abstract

We present a revision of *Glebionis coronaria* in the Mediterranean area based on: a) micro-morphology of the disc floret cypselas observed with a high-resolution confocal microscopy; b) measurements of the disc cypsela with a stereoscopic microscope – duly scaled; c) its distribution in several bioclimatic belts; d) field observations; e) comparisons of herbarium samples. Because of this study, we propose the elevation of Glebionis coronaria var. discolor to the rank of species, as *Glebionis discolor* **comb. & stat. nov.**, based on morphological and ecological characteristics such as the disposition of the intercostal glands, the size of the disc cypsela wings and its distribution according to the bioclimatic belts. *Glebionis coronaria*, with totally yellow ray florets and intercostal glands aligned, is exclusive to the thermo-Mediterranean bioclimatic belt, while *Glebionis discolor*, with white ray florets on a yellow base and intercostal glands arranged randomly, is found in the thermo- and meso-Mediterranean belt.

Illustrations of micromorphological characteristics of the cypselas, an identification key, a taxonomic synopsis including information on nomenclatural types, synonyms, descriptions of the taxa, and, as supplementary information, a list of the specimens examined and bioclimatic classification of samples localities are also presented.

## Introduction

The genus *Glebionis* Cass. ex Spach is present in the Mediterranean area with two species: *Glebionis coronaria* (L.) Cass. ex Spach (= *Chrysanthemum coronarium* L.) and *G. segetum* (L.) Fourr. (= *Chrysanthemum segetum* L.).

For the first species, d’Urville (1822) described the variety with yellow ray florets as Chrysanthemum coronarium var. concolor d’Urv., and the other with white ray florets with a yellow base as C. coronarium var. discolor d’Urv. The only character used by d’Urville to distinguish the two varieties was the colour of the ray florets.

Cassini (1826) gave the first description of the genus *Glebionis* based on the species *Chrysanthemum roxburghii* Desf., and published the new combination *Glebionis coronaria* based on *Chrysanthemum coronarium*, wich was described later by Spach (1841). Subsequently, Pau described a new species under the name of *Chrysanthemum merinoanum* for the island of Ibiza with the following diagnosis: “Intermedio entre el *coronarium* y el *segetum*, pero más afine del primero, del cual difiere por las hojas simplemente pinado-cortadas; los aquenios son muy parecidos, pero carecen de alas tan pronunciadas, y sólo llevan una. ..... lígulas blanquecinas, en la base amarillas, apenas festonadas en la terminación;.....” (Pau 1899). Recently, Rosselló and Sáez (2001) designated a lectotype of *C. merinoanum* Pau (MA 128240) from a specimen collected by Pau on the island of Ibiza, emphasizing that the type material is indistinguishable from other Balearic and Spanish accessions of *C. coronarium* L.

Many authors recognize these two different entities (Fiori 1923, Rechinger 1936, Valdés et al. 1987, Vogt and Aparicio 1999, Bacchetta 2006, Sell 2006, Abd El-Twab et al. 2008, Chilton and Turland 2008, Cano et al. 2012, 2013). Turland (2004) proposes to maintain the name *Chrysanthemum coronarium* L. as the conserved name to designate the type of *Chrysanthemum coronarium* L. [Typus: Greece, Kriti (Crete): Nomos Irakliou, Eparhia Kenourgiou, 500 m E of Gangales, E side of road to Vali (35°03'39"N, 25°00'57"E), 250 m, large field with *Hordeum* crop, 13 Apr 2003, Kyriakopoulos & Turland sub Turland 1166 (UPA; isotypi: B, BM, MO), typ. cons. Humphries (in Jarvis et al., Regnum Veg. 127: 33. 1993)] previously proposed a lectotype of *Chrysanthemum coronarium* after the lectotypification of Dillon (Herb. Clifford: 416, *Chrysanthemum* no. 1, fol. 1 – BM). However, this specimen cannot be used for the lectotypification as it clearly presents ray florets with a darker base.

Turland (l.c.) also confirmed the differentiation of the two varieties and proposed a new combination under the name of Glebionis coronaria var. discolor (d’Urv.) Turland (Basionym: Chrysanthemum coronarium var. discolor d’Urv. in Mém. Soc. Linn. Paris 1: 368. 1822). Turland (l.c.) notes that the two entities appear to be widespread in the Mediterranean region and show no obvious correlation with geographic distribution.

From the karyological point of view the two varieties of *G. coronaria* are both diploid, with 2n = 18 (Pavone et al. 1981 Strother and Watson 1997, Vogt and Aparicio 1999, Inceer and Hayirlioglu-Ayaz 2007, Paciolla et al. 2010, Lograda et al. 2013). Abd El-Twab et al. (2008) confirm this account and point out that the chromosome complement of *G. coronaria* consists of 18 median-centromeric chromosomes, while G. coronaria var. discolor consists of 16 median- and 2 sub-median-centromeric chromosomes.

The aims of this paper were: (a) to highlight and compare some important micromorphological characters of the two entities of *Glebionis coronaria*; (b) to relate their taxonomic differences with their bioclimatic characteristics; (c) to indicate new informative characters for identification of these two taxa; (d) to prepare a key, make a more complete description and provide notes on ecology and distribution of these two entities.

## Methods

### Sampling areas

**Figure 1.**
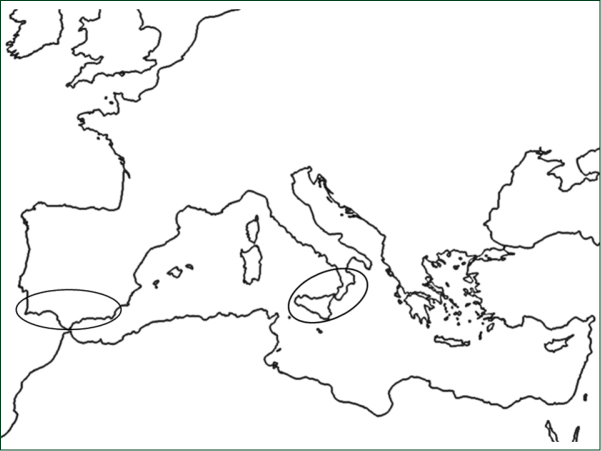
Sampling areas.

To clarify the morphological and ecological characters of the two varieties, we carried out several samplings in different areas of the Mediterranean basin: Sicily, southern Italian Peninsula (Calabria), and Iberian Peninsula (southern Spain and Portugal) (Fig. 1).

The sampling was on bioclimatic criteria and according to the climate classification of Rivas-Martínez and Rivas-Saenz (1996-2009). A statistical analysis was performed with T-Student to establish a possible relationship between the two entities and bioclimatic belts.

### Plant material

A micro- and macro-morphological study was made of sampled plants from pure non mixed populations. All the specimens collected in the field are conserved in the herbaria of Jaén (JAEN) and Reggio Calabria (REGGIO). We have also consulted the following herbaria which have specimens proceeding from eastern Mediterranean regions, the source location of the species originally described by Linnaeus: REGGIO, JAEN, FI, MS, CAT, SEV, VAL, COFC, MA. All 194 examined specimens are listed alphabetically by country in Appendix 1.

Seeds of G. coronaria var. coronaria obtained from pure populations in southern Portugal and Sicily and seeds of G. coronaria var. discolor obtained from pure populations in Jaén (Spain) were cultivated for three years. Both specimens were cultivated in the thermo-Mediterranean town of Andújar (Spain) and in the meso-Mediterranean town of Jaén, where they were grown separately and together to determine their vigour and the permanence of the characters.

High-resolution confocal microscopy was used to study the micro-morphology of the disc floret cypselas. A total of 880 cypselas (322 of the entity with yellow ray florets and 558 of the entity of white ray florets) were measured by taking images with a stereoscopic microscope –duly scaled– of both entities from different populations of plants cultivated in Portugal, Spain and Italy. The measurements were based on several observations ranging from 296 for the variety with yellow ray florets, to 425 for the variety with white ray florets; a statistical treatment was then applied using the XLSTAT programme.

Using these samples, measurements were taken of the length and width of the disc cypselas (excluding ventral and dorsal wings) and the width of the ventral wings (Table 1). We added a measure of the glands dispersion in each cavity formed between the ribs of the disc cypselas. To measure the degree of glands dispersion, a linearity coefficient (Lc) is proposed. A two-pixel wide straight line was drawn on the image between the two most separated glands in length within the group. The glands in contact with the straight line (A) were counted, and these glands were related to all the glands occupying the cavity (T). For cypselas whose morphology was not straight, but whose glands were aligned, two or more lines were used to count the aligned glands, applying a correction factor depending on the number of lines used (C). The formula and its correction are as follows:

Lc= (A-1)/T – (C-1)/A,

where (C-1)/A is the correction factor. If only one line is used, it is = 0.

Lc Linearity coefficient

A Aligned glands

T Number of glands in the valley

C Number of straight lines used

**Table 1.** Disc cypsela measurements of *Glebionis coronaria* and *G. discolor* comb. & stat. nov.

Characters	Parameters	Species	
		*G. coronaria*	*G. discolor*
Wing Width	No. observations	**298**	**425**
	Mean (mm)	**0.741**	**0.557**
	Int. for the mean of 95% (mm)	**(0.719; 0.762)**	**(0.543;0.572)**
	Student’s test p value	**< 0.01**	
	Z test p value	**< 0.01**	
Disc Cypsela Width Without Wing	No. observations	**315**	**425**
	Mean (mm)	**1.960**	**1.932**
	Int. for the mean of 95% (mm)	**(1.905; 2.015)**	**(1.856; 2.007)**
	Student’s test p value	**0.552**	
	Z test p value	**0.552**	
Disc Cypsela Length	No. observations	**313**	**424**
	Mean (mm)	**2.740**	**2.830**
	Int. for the mean of 95% (mm)	**(2.678; 2.803)**	**(2.792; 2.868)**
	Student’s test p value	**0.016**	
	Z test p value	**0.016**	
Linearity Coefficient (Lc)	No. observations	**193**	**356**
	Mean	**0.683**	**0.473**
	Int. for the mean of 95%	**(0.661; 0.706)**	**(0.455; 0.490)**
	Student’s test p value	**< 0.01**	
	Z test p value	**< 0.01**	
Ratio Cypsela-Wing Width	No. observations	**296**	**425**
	Mean	**2.771**	**3.740**
	Int. for the mean of 95%	**(2.676; 2.866)**	**(3.556; 3.923)**
	Student’s test p value	**< 0.01**	
	Z test p value	**< 0.01**	

## Results

To verify the observations made in the field, both varieties (from pure populations in different regions) were cultivated from seeds in the two bioclimatic belts for three years. In the thermo-Mediterranean belt, the seeds of both entities sprouted and produced plants that maintained their characters unchanged from year to year. In the meso-Mediterranean belt both seed entities sprouted initially; however only the white floret variety completed its life cycle and maintained its characters.

**Figure 2.**
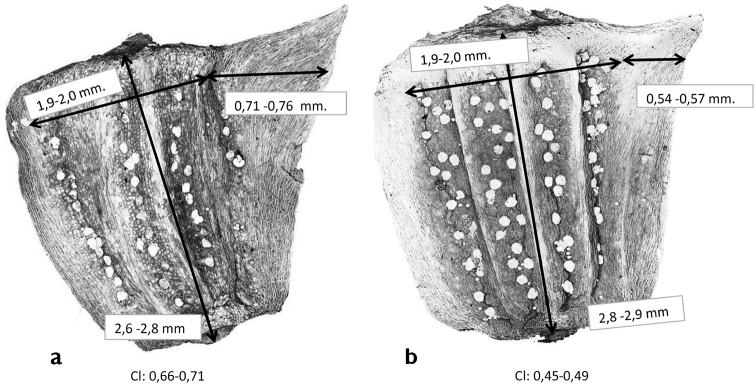
Disc cypsela of *Glebionis coronaria* (**a**) and *G. discolor* (**b**) photographed with high-resolution confocal microscopy.

According to Heywood (1976), sessile non-mucilaginous glands are present between the ribs of cypselas in both varieties. However, after careful observation (Tab. 1), we noticed that in the variety with yellow ray florets these glands were neatly arranged between the ribs (Fig. 2a), while they were disordered in the variety with white ray florets (Fig. 2b).

Other characters that differentiate the two entities are the width and shape of the abaxial wing of the disc floret cypselas. In the yellow floret variety, this wing is wider and the distal tip is facing upward, while in the white floret variety it is narrower and not facing upward (Table 1, Fig. 2a–b).

**Figure 3.**
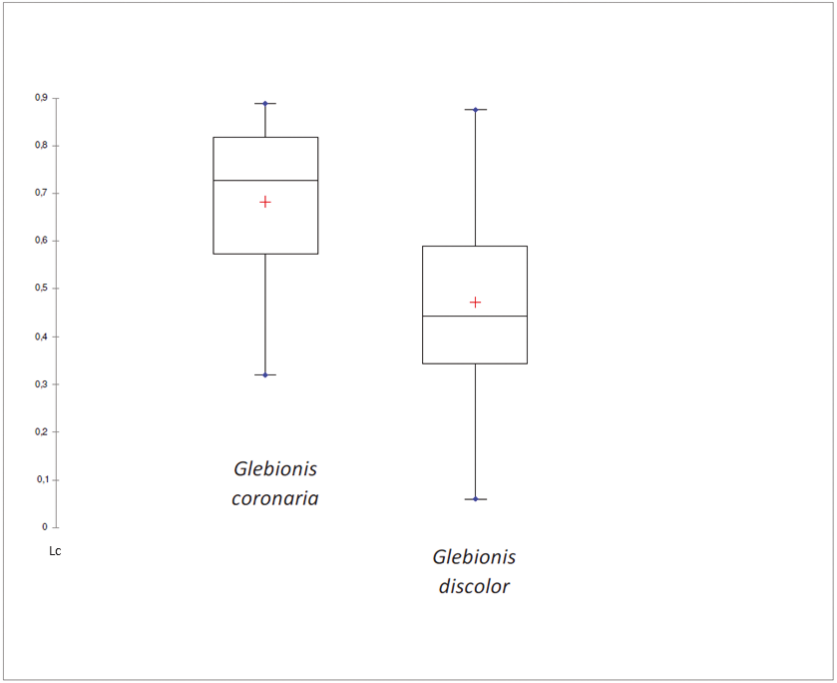
Box plot of alignment of glands distributed along the cypselas of *Glebionis coronaria* and *G. discolor* (Lc = Linearity coefficient).

**Figure 4.**
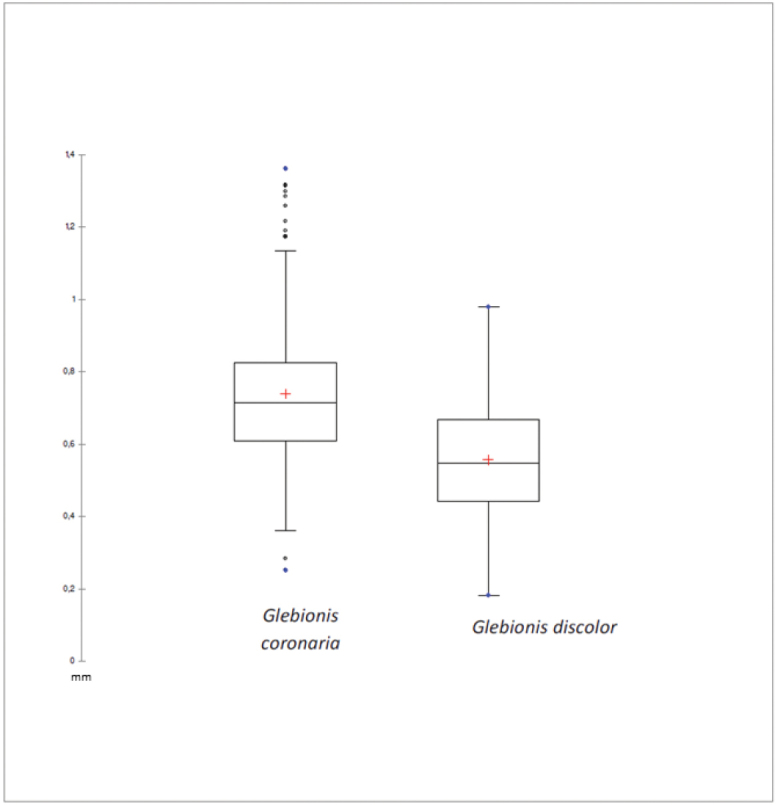
Statistical analysis by box plot of cypselas wing width of *Glebionis coronaria* and *G. discolor*.

Both the arrangement of the glands in the intercostal spaces and the wing width are good characters –among others– for differentiating the two entities, as can be seen from the statistical study (Figs 3, 4). The linearity coefficient was used to measure objectively the arrangement of the glands in the intercostal spaces.

In the boxplot (Fig. 3), the Linearity coefficient of the glands present in the intercostal valleys of the inner cypselas can be observed. In both species, they do not overlap, so it is an important differentiator character: it is therefore that both taxa present morphological differences in the arrangement of the glands.

As for the boxplot analysis of the wing width measurements of the cypsela (Fig. 4), it is observed as this character is also different in both taxa, by not overlapping measures significantly and having a bounded variance.

**Figure 5.**
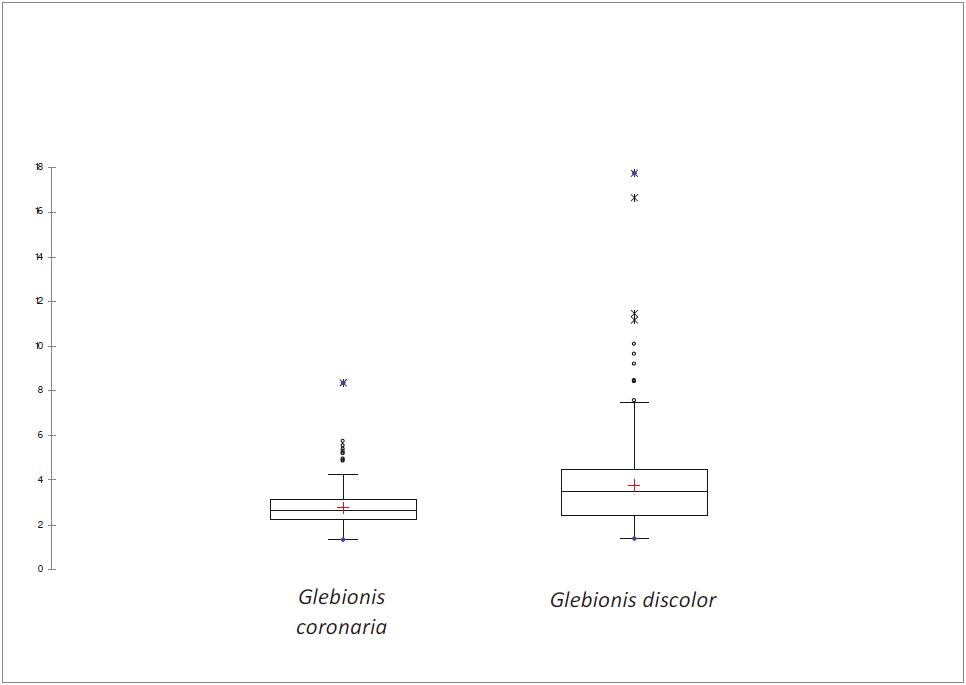
Statistical analysis by box plot of ratio cypsela-wing width of *Glebionis coronaria* and *G. discolor*.

**Figure 6.**
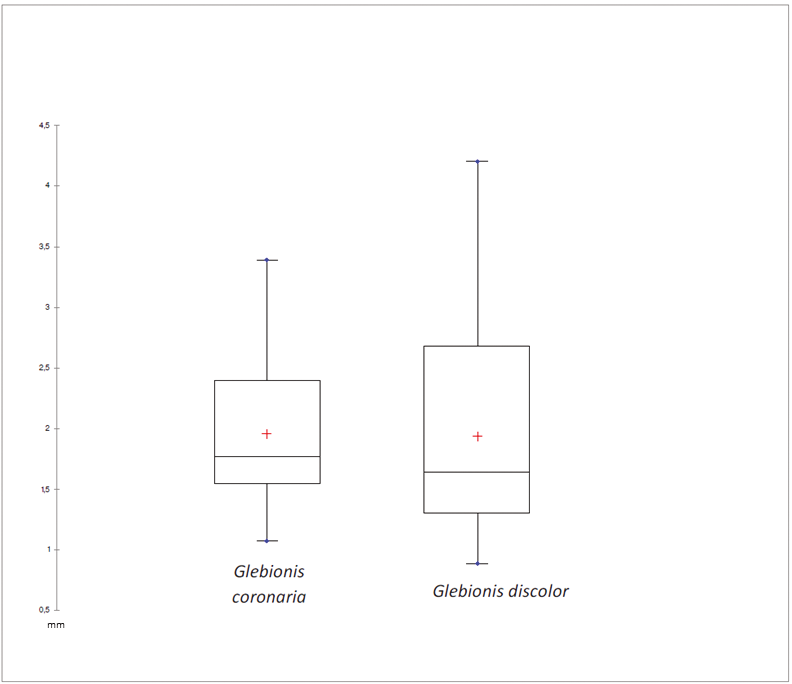
Statistical analysis by box plot of disc cypselas width of *Glebionis coronaria* and *G. discolor*.

**Figure 7.**
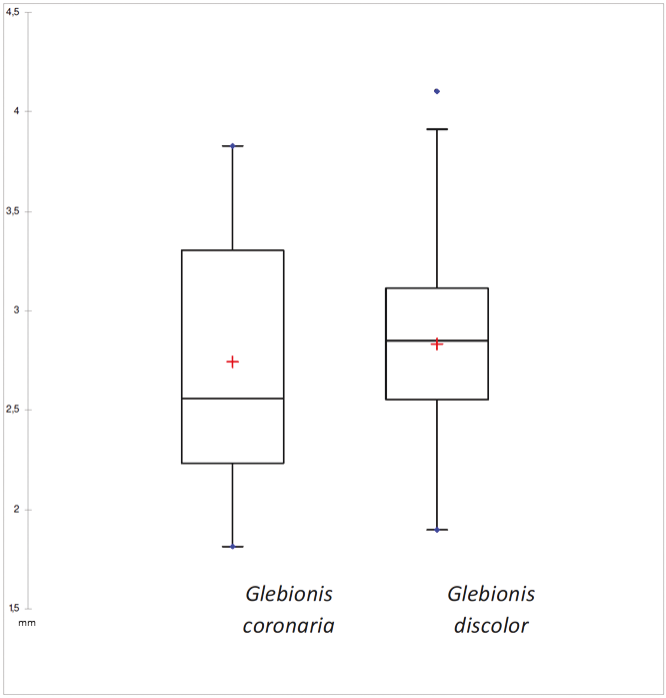
Statistical analysis by box plot of disc cypselas length of *Glebionis coronaria* and *G. discolor*.

However, the ratio cypsela-wing width (Fig. 5), the measures of width (Fig. 6) and length (Fig. 7) of the disc cypselas, are not adequate parameters to differentiate both taxa, since the overlap of the measurements is evident. Although the cypsela length is statistically different between both taxa, as can be seen in Table 1.

An average confidence interval of 95% was used in the statistical treatment. A parametric distribution analysis was applied and gave a P-value with a significance of less than 0.05 in the Student’s T test and the Z test. The margin of error is < 1.62 % in the case of the length of the disc cypsela, and < 0.01% for the arrangement of glands (linearity) and the ratio cypsela-wing width of the disc cypsela (Table 1).

In the analysis of the width of the disc cypsela for the two species, the P value is > 0.05, The character of width and length of disc cypsela therefore does not have much strength in differentiating the species (Figs 6, 7).

**Figure 8.**
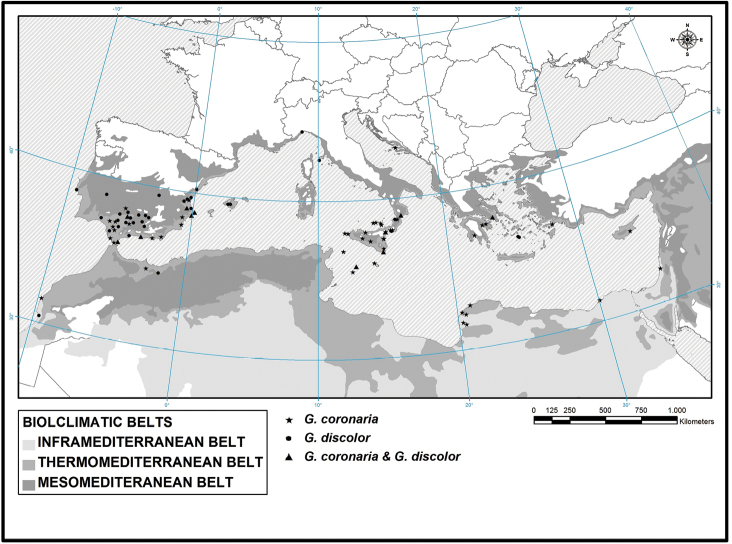
Thermoclimatic distribution of *Glebionis coronaria* (thermo-Mediterranean) and *G. discolor* (thermo and meso-Mediterranean) selected samples studied.

According to the Worldwide Bioclimatic Classification System proposed by Rivas-Martínez and Rivas-Saenz (1996-2009), the localities in which the two *Glebionis coronaria* entities were sampled, fall in two bioclimatic belts: thermo-Mediterranean and meso-Mediterranean [Fig. 8 and Appendix 2].

The thermo-Mediterranean belt is differentiated into the lower (with 400 <Itc <450) and upper thermo-Mediterranean belt (350 <Itc <400). We have collected both *G. coronaria* entities in pure and/or mixed populations in these belts.

Specifically, G. coronaria var. coronaria was sampled in 84% of the 32 stations in the thermo-Mediterranean belt, while G. coronaria var. discolor was sampled in 34% (Table 2).

The two entities are more or less equally distributed in 50 stations in the upper thermo-Mediterranean belt: G. coronaria var. coronaria was sampled in 50% and G. coronaria var. discolor was sampled in 56% (Table 2).

G. coronaria var. coronaria was sampled in 32% of the 25 stations in the lower meso-Mediterranean bioclimatic belt (285 <Itc <350), while G. coronaria var. discolor was sampled in 76% (Table 2).

Only G. coronaria var. discolor was sampled in the single station in the lower meso-temperate belt (Table 2).

**Table 2.** Distribution of *Glebionis coronaria* and *G. discolor* comb. & stat. nov. selected samples studied, related to the different bioclimatic belts.

Bioclimatic belts	N. localities	*G. coronaria*		*G. discolor*		Total n. of samples
		N. of samples	%	N. of samples	%	
Upper Infra-Mediterranean	11	10	91%	2	18%	12
Lower thermo-Mediterranean	32	27	84%	11	34%	38
Upper thermo-Mediterranean	50	25	50%	28	56%	53
Lower meso-Mediterranean	25	8	32%	19	76%	27
Upper meso-Mediterranean	4	1	25%	3	75%	4
Lower meso-Temperate	1	0	0%	1	100%	1
**Total**	**123**	**71**		**64**		**135**

On this basis, the application of the X^2^ test (=0,00247) highlighted the high significance of the preferential distribution of G. coronaria var. coronaria samples in the warmer belts (infra- and lower thermomediterranean), while G. coronaria var. discolor was observed to have a significantly greater presence in cooler belts (meso- and upper thermomediterranean) than G. coronaria var. coronaria.

## Discussion

D’Urville (1822) describes two varieties of *Chrysanthemum coronarium* –*discolor* and *concolor*– taking into consideration only the external female ray floret colour.

Specimens with totally yellow ray florets are now treated as *Glebionis coronaria* (L.) Cass. ex Spach. Also, Turland (2004) considers these two entities as distinct taxa treated at the rank of variety, and proposes a new combination in Glebionis coronaria for var. discolor (Glebionis coronaria var. discolor (d’Urv.) Turland, comb. nov. – Basionym: Chrysanthemum coronarium var. discolor d’Urv. in Mém. Soc. Linn. Paris 1: 368. 1822). This author also maintains that the two varieties may appear in independent or mixed populations, with no difference in distribution. We cannot agree with this author, as our sampling carried out in Sicily, southern Italy, Spain and Portugal, and our observations of specimens from Great Britain (Gibraltar), France, Croatia, Greece, Turkey, Cyprus, Malta, Israel, Egypt, Morocco and Libya reveal that the G. coronaria var. coronaria is distributed exclusively throughout the whole of the thermo-Mediterranean belt with thermo-climatic values of It/Itc = 350–450; while G. coronaria var. discolor is found throughout the thermo- and meso-Mediterranean belt with values of It/Itc =220-350 (Tab. 2), but it is more represented in percentage terms in stations in the meso-Mediterranean belt.

An entity at the specific level of *Chrysanthemum* with bicolour ray florets was previously described by Pau (1899) as *C. merinoanum*. In our opinion, this species is different from Chrysanthemum coronarium var. discolor d’Urville. According to the analysis of the herbarium sample (MA 128240) and from the description given by Pau (1899): “Intermedio entre el *coronarium* y *segetum*…. lígulas blanquecinas...; aquenios calvos, los externos trigonos con una sola ala…”, *C. merinoanum* Pau is a probable hybrid of C. coronarium var. discolor and *C. segetum*. In fact, C. coronarium var. discolor lacks the characters of *C. segetum* and has external cypselas with two wings and two dorsal ribs. *C. merinoanum*, however, has only one wing on the cypsela and leaves that are clearly like those of *C. segetum*.

Moreover, our studies on the morphology of disc cypselas using high-resolution confocal microscopy, morphometric analysis and statistical techniques have revealed sufficient differences to justify raising the variety to a higher rank. Since two subspecies cannot coexist in the same geographic area and even less in the same habitat (criterion of allopatry), we consider them to be two distinct species.

For all these reasons, we propose a lectotypification and a change in rank for Chrysanthemum coronarium var. discolor d’Urville. The two species are listed below, with their differential characteristics highlighted.

## Conclusions

The two entities traditionally included in *Glebionis coronaria* (L.) Cass. ex Spach based on external female ray floret colour have differences in their morphological and ecological features that enable them to be attributed to two different species.

In the study of the material collected in the Mediterranean area, we can confirm that the two varieties given by d’Urville (1822) present major differences in their micro- and macro-morphological characters and their distribution. Moreover, the aforementioned characters of the cypselas are very important for the determination of herbarium specimens, as the colours of the ray florets do not persist when the plants are dried.

Since *Glebionis coronaria* is conserved in the form of plants with yellow ray florets, corresponding to Chrysanthemum coronarium var. concolor d’Urv. and necessarily to G. coronaria var. coronaria, we establish a change of rank for the var. discolor d’Urv. Both entities present clear differences in the colour of their ray florets, the shape and size of their disc cypselas and in the disposition of their glands. For this reason, based strictly on the ICN (McNeill et al. 2012), we maintain the species *Glebionis coronaria* and propose *G. discolor* comb. & stat. nov.

## Taxonomic treatment

### Identification key

**Table:** 

1	Glabrous plant. Female ray florets with completely yellow limb. Disc cypselas 2.6-2.8 mm long, with a pronounced wing (average width 0.71–0.76 mm) and intercostal glands aligned. Species distributed mainly throughout the thermo-Mediterranean bioclimatic belt	***G. coronaria***
–	Plants frequently puberulous. Female ray florets white with a yellow base. Disc cypselas 2.8–2.9 mm long with poorly pronounced wings (average width 0.54–0.57 mm) and intercostal glands arranged randomly. Species distributed throughout the thermo-Mediterranean and meso-Mediterranean bioclimatic belt	***G. discolor***

### Taxonomic synopsis

#### Glebionis coronaria

Taxon classificationPlantaeAsteralesAsteraceae

(L.) Cass. ex Spach, Hist. Nat. Vég. 10: 181. 1841

≡ Chrysanthemum coronarium L., Sp. Pl.: 890. 1753, *nom. cons. ≡ Pyrethrum indicum* Roxb. ex Sims 1813 ≡ Chrysanthemum coronarium var. concolor d’Urv. in Mém. Soc. Linn. Paris 1: 368. 1822 ≡ Chrysanthemum roxburghii Desf. 1829 ≡ Pinardia coronaria (L.) Less., Syn. Gen. Compos.: 255. 1832 ≡ Xanthophthalmum coronarium (L.) P. D. Sell in Sell and Murrell, Fl. Great Britain & Ireland 4: 556, 2006. Typus [by Turland (2004)]: Greece, Kriti (Crete): Nomos Irakliou, Eparhia Kenourgiou, 500 m E of Gangales, E side of road to Vali (35°03'39"N, 25°00'57"E), 250 m, large field with Hordeum crop, 13 Apr 2003, Kyriakopoulos & Turland sub Turland 1166 (UPA; isotypi: B, BM, MO).

##### Note.

*Glabrous plant.* Stems branched, tall 20–80 cm. Leaves semi-amplexicaul, oblong or obovate, 2-pinnatisect with oblong or lanceolate segments. Involucre 10–20 mm long; outer bracts ovate, with brownish marginal bands with a whitish scarious margin; inner bracts without marginal bands but with wider scarious margins. Female ray florets with completely yellow limb. Disc cypselas 2.6–2.8 mm long, with a pronounced wing (average width 0.71–0.76 mm) and intercostal glands aligned (Table 1, Fig. 2a). 2n = 18.

##### Habitat.

Cultivated grounds, along the ways and waste places.

##### Bioclimatic distribution.

Species distributed mainly throughout the thermo-Mediterranean bioclimatic belt.

#### Glebionis discolor

Taxon classificationPlantaeAsteralesAsteraceae

(d’Urv.) Cano, Musarella, Cano-Ortiz, Piñar Fuentes, Spampinato & Pinto Gomes comb. & stat. nov.

urn:lsid:ipni.org:names:77163641-1

Basionym: Chrysanthemum coronarium var. discolor d’Urv. in Mém. Soc. Linn. Paris 1: 368. 1822). ≡ Chrysanthemum coronarium subsp. discolor (d’Urv.) Rech. f. in Beih. Bot. Centralbl. 54B: 634. 1936 ≡ Glebionis coronaria var. discolor (d’Urv.) Turland in Taxon 53: 1073. 2004. Lectotype designated here: Greece, Melos, 05/1819, D’Urville (K 000929476).

##### Note.

Like *G. coronaria* but plants frequently puberulous. Female ray florets white with a yellow base. Disc cypselas 2.8–2.9 mm long, with poorly pronounced wings (average width 0.54–0.57 mm) and intercostal glands arranged randomly (Table 1, Fig. 2b). 2n = 18.

##### Habitat.

Cultivated grounds, along the ways and waste places.

##### Bioclimatic distribution.

Species distributed throughout the thermo-Mediterranean and meso-Mediterranean bioclimatic belt.

## Supplementary Material

XML Treatment for Glebionis coronaria

XML Treatment for Glebionis discolor

## Appendix 1

Selected specimens examined of *Glebionis coronaria*, *G. discolor* comb. & stat. nov. and *Chrysanthemum merinoanum*.

***Glebionis coronaria*** :

- ALGERIA. Hab. in ditione urbis Alger, loco dicto Kouba, 1879, *M. Gandoger n° 499* (FI); recolté aux env. de Bone, Herbages, 21 april 1972 *A. Chabert* (FI); Oran, 1892, *Debaux* (FI).

- CROATIA. Dalmazia – Lesina, marzo 1882, *Marchesetti* (FI); Dalmazia – Pelagosa, marzo 1882, *Marchesetti* (FI).

- CYPRUS. In campis prope Larnaka vetus, Iul. 1880, *Sintenis et Rigo 807* (FI); Ayia-Anna (Larnaca), Altim. 150 m, bords de culture sur substrat de calcaires et marnes du Paléogène, 15-04-1991, *Alziar et al.* (FI); entre Xylophagou et Ayia Thekla (Larnaca), altim. 5m, champ (blé) abandonné, et pseudosteppe à *Sarcopoterium*, 12-IV-1991, *Alziar et al.* (FI).

- EGYPT. Ramle presso Alessandria, marzo 1898, *Marchesetti* (FI); ….del porto di Alessandria che segue il litorale della regione di Ramle ed Abuhir, maggio 1867, *Figari* (FI); barbus field, sandy soil, Burg El Arabi, W. Medit Coast. 30.3.1957,… (FI).

- FRANCE. Pyrenées, in cultis, marzo 1848, *Franqueville* (FI).

- GREAT BRITAIN. **Gibraltar**: Catalan Bay, arenas mezcladas con rocas calizas, 16 may 1985, *J. Bensusan, S. Talavera, B. Valdés 124741* (SEV).

- GREECE. Kriti (Crete), Nomos Irakliou, Eparhia Kenourgiou, 500 m E of Gangales, E side of road to Vali (35°03'39"N, 25°00'57"E), 250 m, large field with Hordeum crop, 13 Apr 2003, Kyriakopoulos & Turland sub Turland 1166 (MO 5792988); In ruderatis, ad vias Graeciae, Athenis 8 april 1852, *Heldreich* (FI). Rodi-Egeo, San Giovanni, 1934, … (FI).

- ISRAEL. Ramath-Gan, near Tel-Aviv, field borders, 12.IV.1928, *N. Feinbrun, L. Schachnowitz et D. Soltchansky* (FI).

- ITALY. **Calabria**: Torrente Fiumarella, Pellaro (Reggio Calabria), 11m, 38°01'14,89"N, 15°38'42.95"E, 19 May 2012, *C.M. Musarella, 4179/1-2-3-4* (REGGIO); Torrente Fiumarella, Pellaro (Reggio Calabria), 11m, 38°01'14,89"N, 15°38'42.95"E, 19 May 2012, *C.M. Musarella, 130106-130107-130108* (JAEN); S. Nicola da Crissa, Serre (Vibo Valentia), 10 June 2005, *Spampinato G.* 2331 (REGGIO); SP 3 km 48, 741 m slm, sopra Bagaladi (Reggio Calabria), 23 May 2013, *Cano, Musarella, Mendoza, Piñar-Fuentes* 4181; Catanzaro, nei campi lungo le siepi e i sentieri sotto Bellavista (fondo Tubolo, proprietà Arbitrio), maggio-giugno 1895, *L. Micheletti* (FI); dintorni di Catanzaro, 4/6/1883, *A. Fiori* (FI); Catanzaro, lungo le siepi e i sentieri sotto Bellavista, maggio 1895, *L. Micheletti* (FI). **Sicily**: Monte Kalfa (Messina), 24 February 2001, *A. D’Arrigo 000896* (MS); M. Grasso (Siracusa), 08 April 1989, *Bartolo G., Pulvirenti S. 3002* (CAT); Fiume Ferro (Catania), 01 June 1985, *Spampinato G. 3003* (CAT); Piana di Catania, 01 August 1957, ......... *3004* (CAT); Lipari (Isole Eolie, Messina), 27 April 1982, *Brullo S. 3008* (CAT); Lampedusa (Isole Pelagie, Agrigento), 18 March 1985, *Brullo S., Minissale P., Spampinato G. 3009* (CAT); M. Mela (Agrigento), 25 April 1969, *Brullo S. 3010* (CAT); Isole Eolie, Messina), 31 May 1980, *Brullo S. 3011* (CAT); Alicudi (Lipari, Cava di Pomice (Isole Eolie, Messina), 13 May 1972, *Brullo S. 3012* (CAT); da Piano Conte alla Terme di San Calogero (Isole Eolie, Messina), 28 May 1969, *Furnari F. 3013* (CAT); Filicudi (Isole Eolie, Messina), 30 April 1980, *Brullo S. 3014* (CAT); Termini Imerese, Fiume Imera (Palermo), 27 April 1983, *Brullo S. 3015* (CAT); Pantano Longarini, Pozzallo (Ragusa), 25 April 1969, *Brullo S. 3017* (CAT); Favignana (Isole Egadi, Trapani), 14 April 1973, *Brullo S. 3018* (CAT); Noto (Siracusa), 16 May 1980, *Brullo S. 3019* (CAT); Linosa, ad oras et in culti (ligulae concolores), 24 aprili 1873, *S. Sommier* (FI); Insula Linosa (olim Aethusa) prope portum, 1 Martii 1906 legi, *Stephen Sommier* (FI); Insula Lampedusa (olim Lopadusa) prope portum vulgata, 08 martii 1906 legi, *Stephen Sommier* (FI); Insula Pantelleria (olim Cossyra), Alle Balate, In insula vulgata, 16 Martii 1906 legi, *Stephen Sommier* (FI); Caltanissetta, IV 1893, *A. Fiori 03* (FI); Insula Linosa (olim Aethusa) prope paguis, 1 Martii 1906 legi, *Stephen Sommier* (FI); Marettimo, gita dal faro a Capo Troja, 27/04/1935, , (FI). **Sardinia**: Quartu Sant’Elena, San Forzorio sponda E dello Stagno di Simbirizzi, 19 Maggio 1065, *G. Martinoli, T. Onnis* (FI).

- LIBYA. **Cyrenaica**: Tolmeta, 17 March 1975, *Brullo S., Furnari F. 3022* (CAT); Spiaggia Sini Bu Giarrar, 20 March 1974, *Brullo S., Furnari F. 3023* (CAT); Scavi di Tolmeta, 11 May 1974, *Brullo S., Furnari F. 3024* (CAT); Driana, 31 March 1974, *Brullo S., Furnari F. 3025* (CAT); Tolmeta, scavi, 28 March 1974, *Brullo S., Furnari F. 3026* (CAT); Tolmeta, 9 March 1975, *Brullo S., Furnari F. 3028* (CAT); Zona alta di Wadi el – Bab, 05 March 1982, *Furnari F., Signorello P. 3029* (CAT); Driana, 28 March1981, *Brullo S., Furnari F. 3031* (CAT); Tolmeta, 11 March 1974, *Brullo S., Furnari F. 3032* (CAT); Tolmeta Scavi, 24 March 1974, *Brullo S., Furnari F. 3033* (CAT); Sebelet Bu Giarrar, 20 March 1974, *Brullo S., Furnari F. 3034* (CAT); El Abiar, 01 April 1974, *Brullo S., Furnari F. 3035* (CAT); Got el Gein, 06 May 1974, *Brullo S., Furnari F. 3036* (CAT); Tocra, 01 April 1974, *Brullo S., Furnari F. 3042* (CAT); Piana di Soluq – ana, 10 March 1982, *Furnari F., Signorello P. 3045* (CAT); Rabiat al Magur (Msus), 02 April 1981, *Brullo S., Furnari F. 3046* (CAT); Sebelet el Cuz, 03 April 1974, *Brullo S., Furnari F. 3041* (CAT); Carrhuba, 01 April 1975, *Brullo S., Furnari F. 3044* (CAT).

- MALTA. **Comino**: Insula Comino, 24 aprilis 1907 legi, *Stephen Sommier n° 390* (FI); Melitae in campis et agris, agosto 1848, *G.C, Grech-Delicata* (FI); Insula Gaulos, 4/8/1874, *J.F. Duthie* (FI);

- MOROCCO. Nador, Plain de Gereb, 36 Km from Selouane, road to Mechrâ-Homadi, Marls and limestones, slopes and dried river bed, 31 May 1993, 400m., 34°50'N 2°52'W, *M. A. Mateos et B. Valdés 150086* (SEV); entre Mogador et Maroc, Ibrahim, 1883, *Cosson* (FI).

- PORTUGAL. In insula Azorre, marzo 1838, *Guthnick* (FI); communicated from the Island of Terceira, Azores, by T.C. Hunt, Esq., British Consul., 8-1846, *A. Chabert* (FI); Albufeira-Gralheira, por entre as rochas calcárias, 23/4/1968, *I. Nogueira* (FI).

- SPAIN. **Andalucía**: Almuñecar, en cunetas y bordes de camino y carreteras, a la salida de la población (Granada), 5m, 30SVF47, 28 May 1982, *Marín Calderón y Hurtado 851975* (JAEN); Almuñeca Punta de la Mona (Granada), 10 m, 6 June 1974, *C. Fernandez Lopez 74-1621* (JAEN); alrededores de Alcalá de Guadaira (Sevilla), 6 March 1975, *R. de Clavijo 64849* (SEV); Alrededores Puente Genil, Entre Puente Genil, Jauja, Río Anzur (Cordoba), 18 April 1980, *Díaz et Muñoz 63449* (SEV); Tarifa, E. side of causeway (Cadiz), 27 March 1969, *V. H. Heywood, D. M. Moore et al. 13695* (SEV); Las Cabezas, Autopista Sevilla-Cádiz (Sevilla), 30 April 1979, *E. F. Galiano, A. Ramos et E. Elvira 63744* (SEV); Conquero (Huesca), 12 May 1979, *P. Romero et al. 52401* (SEV); entre Arcos y Bornos. Carretera a la presa del pantano de Bornos. Bajura, tierras de aluvión (Cadiz), 50-100 m, TF75, 9 May 1980, *A. Martínez 63743* (SEV); Vejer (Cadiz), 4 May 1969, *E. F. Galiano, Gibbs, S. Silvestre et B. Valdés 63597* (SEV); entre Pilas y Aznalcázar (Sevilla), 9 April 1966, *64004* (SEV); El Gandul (Sevilla), 24 March 1969, *E. F. Galiano, et B. Valdés 63595* (SEV); Almería, el Ejido, márgenes carretera, 17 March 1984, *G. Mateo & R. Lázaro VAL50139* (VAL); Almería, 5 Km ESE of Campohermoso, 1.5 Km, N of Fernán Pérez, 180m, 36°55'N 2°5'W, 30S 582200 4086900 (VAL); Almodóvar del Río, desmbocadura del río Guadiato (Córdoba), 2 June 1981, *F. Infante, J. A. Varela 32699* (COFC); Valle del Guadiato, puente de la Cabrilla (Córdoba), 24 April 2010, *C. Granados 51013* (COFC); Xabia Almodóvar del Río, desembocadura del río Guadiato (Córdoba), 24 April 2010, *C. Granados 32702* (COFC); Puente Genil, Cortijo de “Tíscar”, Laguna salada, margen derecho del río Genil (Córdoba), 24 April 1981, *F. Infante, E. Hernández 35467 1/2* (COFC). **Comunidad Valenciana**: Altea – Marina Baixa (Alicante), desembocadura Riu Algar, 5m, 30SYH577720 May 2008, *Aguilella, Torres, Lluzar, Sanchez & Moreno VAL193969* (VAL); Xátiva (Costera), Riu Canyoles (Valencia), 30SYJ1627, 3 March 2010, *C. Torres, E. Lluzar VAL202586* (VAL); Xátiva (Costera), Riu Canyoles (Valencia), 30SYJ1622, 15 April 2010, *C. Torres Gómez, E. Lluzar VAL202534* (VAL); Paiporta (Horta), L´Horta (Valencia), herbazales nitrófilos em campos abandonados, 20m. 30SYJ226652, 28.IV.2011, *S. Fos VAL205853* (VAL); Novelda (Alicante) 14.IV.1933, *C. Pau & E. Moroder VAL161408* (VAL); S-facing limestone bank at edge of field, 17 April 1994, *S. L. Jury n°14685 VAL 143065* (VAL); Marines, Viejo (Valencia), monte, 450m, YK10, 18 April 1999, *L. Moratalla VAL108096* (VAL); Catarroja, Puerto (Valencia), Borde de un camino, 28 February 1998, *Daniel Ballesteros Bargues 105636* (VAL); Dehesa de campamor (Alicante), XG99, 8 February 1987, *G. Mateo et col. VAL70611* (VAL); Paterna (Valencia), herbazal nitrófilo en cuneta de carretera, 50m. 30SYJ2375, 9 April 1992, *J. Cuchillo VAL85417* (VAL).

- TURKEY. Caria, 1843, *Pinard* (FI)

***Glebionis discolor*** :

- ALGERIA. Hab. in ditione urbis Alger, loco dicto Kouba, 1879, *M. Gandoger n° 489* (FI); environs d’Oran, dans les cultures, 28 mars 1913, *A.F.* (FI); Penez, 1891, *Debaux* (FI).

- GREAT BRITAIN. **Gibraltar**: Catalan Bay, arenas mezcladas con rocas calizas, 16 May 1985, *J. Bensusan, S. Talavera, B. Valdés 124741* (SEV).

- GREECE. Greece, Melor, 05/1819, D’Urville (K 000929476); Isole dell’Egeo – Lero, maggio 1938, *T. Colonnello Pietro Bertoglio* (FI); Isola di Rodi: fra Villanova e Fanez, 4 maggio 1922, *N. Mazzocchi-Alemanni* (FI); Dodecanneso: Chefalo, 1-VIII-1924, *A. Desio* (FI); Isola di Rodi: Cattavia, 26 aprile 1922, *N. Mazzocchi-Alemanni* (FI); Isola di Rodi: dintorni di Rodi, 15-30 aprile 1922, *N. Mazzocchi-Alemanni* (FI); Rodi – Dintorni, 17.V.1912 e 9.2.914, … (FI); Isola di Rodi: Lindos, 25 aprile 1922, *N. Mazzocchi-Alemanni* (FI); Isola di Rodi (Egeo), Rodi, 5 agosto 1923, *A. Fiori* (FI).

- ITALY. **Calabria**: Torrente Fiumarella, Pellaro (Reggio Calabria), 10m, 38°01'15.72"N, 15°38'42.00"E, 19 May 2012, *C.M. Musarella 130101-130102* (JAEN); Torrente Fiumarella, Pellaro (Reggio Calabria), 10m, 38°01'15.72"N, 15°38'42.00"E, 19 May 2012, *C.M. Musarella 4178/1-2-3* (REGGIO); Pentedattilo (Reggio Calabria), 24 April 2000, *C.M. Musarella 000240* (MS); Catanzaro, sotto Bellavista, giugno 1895, *L. Micheletti* (FI); Catanzaro, sotto Bellavista, nei ruderi e lungo le siepi, maggio 1895, *L. Micheletti* (FI). **Sicilia**: Monte Kalfa (Messina), 24 February 2001, *A. D’Arrigo 000897* (MS); Pantano Bruno, Pozzallo (Ragusa), 25 April 1969, *Brullo S. 3021* (CAT); Insula Linosa (olim Aethusa) prope paguis, 1 Martii 1906 legi, *Stephen Sommier* (FI); Insula Linosa (olim Aethusa) prope paguis, 2 Martii 1906 legi, *Stephen Sommier* (FI); In aris Linosa, IV/1905, *L. Micheletti* (FI); In cultis Lampedusa…, IV 1905, *G. Zodda* (FI). **Liguria**: Varazze, 24 V 1929, Gavioli 15606 (FI); **Toscana**: Insula Pianosa (olim Planasia vel Planaria), al Marchese – prima l’abitato ……18-19/5/1909 legi, Stéphen Sommier (FI).

- LIBYA. **Cyrenaica**: Soluch a sud di Bengasi, 10 mar. 1933, *R. Pampanini 8582* (FI); Sirual Zauiet el-Hamama, 29 mar. 1933 *R. Pampanini 8583* (FI); Cirene, 18 aprile 1933, *R. Pampanini 8584* (FI); ez-Zuetina a nord-est di Agedabia, 11 aprile 1934, *R. Pampanini e R. Pichi-Sermolli 8586* (FI); Chaulan, 20 aprile 1934, *R. Pampanini e R. Pichi-Sermolli 8587* (FI).

- MOROCCO. Agadir, prope oppidulum Tafraute, Tizi Mlil, in rupestribus siliceis, 9362, 1600m, 29°45'N, 8°52'W, 26 May 1985, *C. Blanché, J. Fernández Casas, J. Molero, J. M. Montserrat & A. Romo 123837* (SEV); Oujda, 6 Km from Oujda in the road to Taza, Calcareous soils, 550m, 34°41'N 1°59'W, 29.V.1993, *M. Etlaftski, M. A. Mateos & B. Valdés 150060* (SEV); in lapidosis arenaris prope Goulimine, 400-500m, 13 aprilis 1935, *E. Wilczek* (FI).

- PORTUGAL. S. Pedro do Estoril proximo da ribera do Caparide (Lisboa), 21 May 1978, *L. A. Grandvaux Barbosa 121257* (SEV).

- SPAIN. **Andalucía**: El Zumbel (Jaén), 600m, UVG-37, 10 April 1974, 74-1999 (JAEN); Linares, ciudad camino (Jaén), 430m, 30SVH4415J, 12 March 1992, *García Rosa 921968* (JAEN); Jabalquinto (Jaén), alrededores margo-calizas, 480m, 30SVH3608, 02 May 1994, *B. Lendinez (Etnobotánica) 940730* (JAEN); Lopera (Jaén), alrededores, 300m, 30SUH0093, 15 May 1995, *Concepción Alcalá Sanz 950606* (JAEN); Baeza, puente Mazuecos, El Guadalquivir Aluvi (Jaén), 300m, 30SVG6098, 22 May 1994, *M.A. Espinosa et B. Chica 944365* (JAEN); cerca de Cerro Molina (Jaén), margas y calizas, 440m, 30SVG3582, 11 May 1991, *A. Gonzalez 910615* (JAEN); Jaén, Puente Jontolla (Jaén), margas calizas, 440m, 30SVG3480, 28 March 1991, *A. Gonzalez 910271* (JAEN); Carretera Las Infantas (Jaén), margas calizas, 360m, 30SVG3291, 10 July 1998, *D. Casado Ponce 980649* (JAEN); Los Yesares (Jaén), margas y yesos, 480m, 30SVG3583, 19 May 1991, *A. Gonzalez 910643* (JAEN); Mengibar, orilla del Guadalquivir (Jaén), 240m, 30SVH3004, 08 May 1982, *E. García Hernandez 82943* (JAEN); Jaén, C. Tallán – 400m, VG-38, 13 May 1981, *C. Fernandez Lopez, M. Portela, L. Morillas 812177* (JAEN); Úbeda, El Donadio, Cerro de los Valencia (Jaén), 400m, 30SVG6898, 17 May 1996, *M.A. Espinosa, C. Fernandez, A. Camacho 960368* (JAEN); Arjona hacia Porcuna, arroyo margas calizas (Jaén), 380m, 30SVG0297, 26 March 1997, *D. Casado 970606* (JAEN); Porcuna San Pantaleón (Jaén), margas calizas, 380m, 30SUG8893, 25 January 2002, *D. Casado Ponce 620138* (JAEN); Marmolejo, Los Miñones (Jaén), terrenos siliceos, 300m, 30SUH9115, 20 April 1984, *E. Cano 844080* (JAEN); Andújar, proximidades del casco Mirasierra (Jaén), 212m, 18 April 2012, *E. Cano 130113* (JAEN); Andújar, proximidades del casco Mirasierra (Jaén), 212m, 18 April 2012, *E. Cano 4180* (REGGIO); Mengíbar, Orilla del Río Guadalquivir (Jaén), 240m, 30S VH 3004, 8 May 1982, *E. García Hernández VAL151608* (VAL); entre Morón y Montellano (Sevilla), 15 April 1977, *E. Ruiz de Clavijo 29165* (SEV); entre Morón y Montellano (Sevilla), 29 April 1977, *E. Ruiz de Clavijo 29026* (SEV); entre Écija y Herrera (Sevilla), cunetas, 5 April 1977, *B. Cabezudo, S. Talavera et al. 63598* (SEV); Carretera Lora – Constantina (Sevilla); 12 April 1981, *P. Escalza, M. López et R. Luque 64151* (SEV); Morón de la Frontera (Sevilla), zona arvense, 26 February 1978, *M. R. Guerrero Cabezas et I. Fernández 63451* (SEV); entre novelda Motril y Almuñecar (Granada), 21 May 1971, *E. F. Galiano, E. Paunero, S. Silvestre et B. Valdés 8399* (SEV); *Chrysanthemum coronarium* L – Corral et Fernández, 31.VII.1980, 64005 (SEV); Camino del canal de riego que desemboca en la carretera de Córdoba a Sevilla, S. Talavera; Pinar y Arroyo Guadalbaida (Córdoba), 23 May 1980, *Fernández 63745* (SEV); Jerez, Salida hacia Sanlúcar (Cadiz), 9 March 1978, *J. Pastor, S. Talavera et B. Valdés 63596* (SEV); entre el Viso del Alcor y Carmona (Sevilla),13 April 1975, *E. Ruiz de Clavijo 66249* (SEV); entre Écija y Herrera, Santa María de la Gracia: Río Genil (Sevilla), 25 June 1986, *C. López et C. Romero 157848* (SEV); Almuñécar (Granada), em cunetas y bordes de caminos y careteras, a la salida de la población, 5m. 30SVF47, 28 May 1982, *Marín Calderón y Hurtado VAL70610* (VAL); Arroyo salado, carretera Montilla-Montalbán (Córdoba), VG-56, 1 April 1985, *F. García 18820*; carretera Santaella-Montalbán (Córdoba), limite oeste de la Teris, 22 May 1985, *F. García 18856*; arroyo Guadalora, Estación de Hornachuleos (Córdoba), carretera de Hornachuelos Km1, 18 April 1980, *P. Fernández, I. Porras 17163*; Río Anzur, Cortijo los Davales (Córdoba), VG-53, 18 April 1985, *F. García 18900*; Río Lucena (Córdoba), entre Moriles y los “Pedros”, VG-54, 15 April 1985, *F. García 18901*; cruce del Bembezar con arroyo Guadalora, Carretera de Hornachuelos (Córdoba), 8 April 1979, *L. Corral, P. Fernández 17160*; carretera Córdoba-Sevilla, Km 40 (Córdoba), 27 January 1980, *P. Fernández, I. Porras 17736*; camino vecinal Hornachuelos-Villaviciosa de Córdoba (Córdoba), cruce con carretera al pantano Bembezar, 1 May 1980, *L. Corral, P. Fernández 17739*; arroyo de Guadalbaida, Cerro Gordo, Tramo de las Posadas (Córdoba), 23.V.1980, *P. Fernández, I. Porras 17742*; Hornachuelos, Fuente del Caño de Hierro (Córdoba), 18 March 1981, *P. Fernández, I. Porras 17734*; valle del Guadiato, puente de la Cabrilla (Córdoba), 4 March 2008, *C. Lucena 51013*; Facultad de Ciencias, Avda San Alberto Magno (Córdoba), 20 March 1990, *E. Ruiz de Calvijo 55070*; Almodóvar del Río, desmbocadura del río Guadiato (Córdoba), 5 April 1981, *J. A. Varela 32701 1/2*; Almodóvar del Río, desmbocadura del río Guadiato (Córdoba), 5 April 1981, *J. A. Varela 32701 2/2*; Alahurín de la Torre (Málaga), finca Ana María La Baja, M. Royo, 24 March 2011, *C. Granados, M. Royo, J. L. Ubera 59911*; **Aragona**: Pilas, Cortijo Hato-Ratón, dehesa, arenas (Huesca), 8 May 1995, *E. Moreno, M. E. Ocaña, M. Parra 136034* (SEV); Gibraleón, cercanías (Huesca), 17.V.1979, *S. Silvestre et S. Talavera 63742* (SEV); **Balearic Islands**: Mallorca, Algaida, alrededores del pueblo em direccion a Monturi, em borde de camino, 39°34'N 2°54'E, 31SDD9079, 2 June 1996, *C. Aedo, N. López, R. Morales, C. Navarro, Ll. Sáez & M. Velayos.........* (VAL); Illes Balears, Mallorca, Campos, caminos, 4 May 1952, *P. Ferrer VAL158014* (VAL); **Castilla-La Mancha**: Mota del Cuervo, Monte Gila (Cuenca), Ladera de um pequeño montículo, 710m, 30SWJ1374, 21 May 2000, *V. Hernaz VAL140930* (VAL); **Comunidad Valenciana**: Altea, Playa de L´Albir (Alicante) en *Resedo-Chrysanthemetum coronarii* O. Bolós & R. Molinier, 7 April 1984, *G. Stübing & J. B. Peris 110918* (SEV); La Nucia, La Marina Baixa (Alicante), pr. poble, 30SYH5078, 19 March 2008, *C. Torres, E. Lluzar VAL189752* (VAL); Xátiva, Costera, Riu Canyoles (Valencia), 15 April 2010, 30SYJ1622C, *Torres Gómez, E. Lluzar.......* (VAL); Riba-roja, Camp de Túria (Valencia), Entrepins herbazal sobre vertidos, 90m, 30SYJ1380, 5 April 2001, *A. Peña VAL205419* (VAL); Castelló de la Plana, La Plana Alta Platija del Grau (Castellón), 2m, 31TBE43, 2 June 1989, *J. Tirado & C. Villaescusa VAL20109* (VAL); Oropesa, La Plana Alta, Camp de Batalla (Castellón), 100m, 31TBE5, 24 May 1993, *A. Aguilella & J. Tirado VAL28220* (VAL); Xabia, Marina Alta (Alicante), vora camins, 1 May 1992, *L. García VAL27571* (VAL); Bunyol, La Foia de Bunyol, Carcalín (Valencia), herbazales subnitrófilos, 400m, 30 SXJ 86, 24 April 1997, *J. Riera VAL40721* (VAL); Yávota, collado de Montratón (Valencia), herbazal de camino, 400m, XJ8660, 13 April 1996, *C. Macián VAL99065* (VAL); El Pla, Carcaixent (Valencia), YJ22, 23 April 1986, *S.Pierra VAL54641*; campo de cereal por la Valleta d´Agres, El Condat (Alicante), 550m, YH19, 6 June 1988, *J. A. Nebot VAL66835* (VAL); Sagunto (Valencia), playa Almardá, arenal costero, 0m, YJ3997, 1 May 1991, *A. García VAL75769* (VAL); **Extremadura**: Badajoz, Pâturages nitrophiloes (*Anacyclo radiati-Hordeetum leporini*), 21 May 1973, *S. Rivas Goday et S. Rivas-Martínezbaez*

***Chrysanthemum merinoanum*** :

- SPAIN. **Balearic Islands**: Ibiza (Baleares) in campis, IV 1899, *Pau 128240* (MA).

## Appendix 2

**Table:** Bioclimatic classification of samples localities and realted distribution of *Glebionis coronaria* and *G. discolor* comb. & stat. nov.

Locality	*Glebionis coronaria*	*Glebionis discolor*	Climate	Ombrotype
Abuhir	x		Lower Mesomediterranean	Upper Arid
Agadir		x	Upper Inframediterranean	Lower Semiarid
Alahurín de la Torre		x	Lower Thermomediterranean	Lower Dry
Alcalá de Guadaira	x		Upper Thermomediterranean	Lower Dry
Alicudi	x		Lower Thermomediterranean	Lower Dry
Almería 5 km Campohermoso	x		Lower Thermomediterranean	Lower Semiarid
Almería el Ejido	x		Lower Thermomediterranean	Lower Semiarid
Almodóvar del río	x	x	Upper Thermomediterranean	Upper Dry
Almuñécar	x	x	Lower Thermomediterranean	Lower Dry
Alrededores Puente Genil	x		Upper Thermomediterranean	Upper Dry
Altea	x	x	Lower Thermomediterranean	Lower Dry
Arjona hacia Porcuna		x	Lower Mesomediterranean	Lower Dry
Arroyo de Guadalbaida posadas		x	Upper Thermomediterranean	Upper Dry
Arroyo salado		x	Lower Mesomediterranean	Upper Dry
Atenas	x		Upper Thermomediterranean	Upper Semiarid
Atenas (acropolis)	x	x	Upper Thermomediterranean	Upper Semiarid
Badajoz		x	Lower Mesomediterranean	Lower Dry
Baeza Puente Mazuecos		x	Lower Mesomediterranean	Upper Dry
Bagaladi	x		Lower Mesomediterranean	Lower Subhumid
Bu giarrar	x		Upper Inframediterranean	Lower Semiarid
Caltanissetta	x		Lower Mesomediterranean	Lower Dry
Caria	x		Upper Thermomediterranean	Lower Subhumid
Carretea Santaella-Montalbán		x	Upper Thermomediterranean	Upper Dry
Carretera Córdoba-Sevilla		x	Upper Thermomediterranean	Upper Dry
Carretera las infantas		x	Upper Thermomediterranean	Lower Dry
Carretera Lora û constantina		x	Upper Thermomediterranean	Upper Dry
Castelló de la plana		x	Upper Thermomediterranean	Lower Dry
Catalan bay	x	x	Lower Thermomediterranean	Lower Subhumid
Catanzaro	x	x	Lower Mesomediterranean	Lower Subhumid
Catanzaro	x	x	Lower Mesomediterranean	Lower Subhumid
Catarroja	x		Lower Thermomediterranean	Lower Dry
Cerca de Cerro Molina (Úbeda)		x	Lower Mesomediterranean	Lower Dry
Cossyra	x		Lower Thermomediterranean	Lower Dry
Cruce del Bembezar con arroyo Guadalora		x	Upper Thermomediterranean	Upper Dry
Dehesa de Campamor	x		Upper Thermomediterranean	Lower Semiarid
Driana	x		Upper Inframediterranean	Lower Semiarid
El Abiar	x		Upper Thermomediterranean	Upper Arid
El Gandul (Se)	x		Upper Thermomediterranean	Lower Dry
El Pla Carcaixent		x	Upper Thermomediterranean	Lower Dry
El Zumbel		x	Upper Mesomediterranean	Upper Dry
Entre Arcos y Bornos.	x		Upper Thermomediterranean	Upper Dry
Entre Écija y Herrera		x	Upper Thermomediterranean	Lower Dry
Entre el Viso del Alcor y Carmona		x	Upper Thermomediterranean	Upper Dry
Entre Morón y Montellano		x	Upper Thermomediterranean	Upper Dry
Entre Motril y Almuñecar		x	Lower Thermomediterranean	Lower Semiarid
Entre Pilas y Aznalcázar	x		Upper Thermomediterranean	Lower Dry
Epidaura	x		Upper Mesomediterranean	Lower Subhumid
Estación de Hornachuleos		x	Upper Thermomediterranean	Upper Dry
Favignana	x		Lower Thermomediterranean	Lower Dry
Filicudi	x		Lower Thermomediterranean	Lower Dry
Fiume Ferro (Ct),	x		Upper Thermomediterranean	Lower Dry
Gibraleón cercanías		x	Upper Thermomediterranean	Lower Dry
Gozo	x		Lower Thermomediterranean	Lower Dry
Hornachuleos		x	Upper Thermomediterranean	Upper Dry
Illes Balears Mallorca		x	Lower Thermomediterranean	Upper Semiarid
Insula Comino	x		Lower Thermomediterranean	Lower Dry
Insula Linosa (Aethusa)	x	x	Lower Thermomediterranean	Lower Semiarid
Insula Pianosa		x	Upper Thermomediterranean	Lower Dry
Isole Eolie (Me)	x		Upper Thermomediterranean	Upper Dry
Jabalquinto (Jaén)		x	Lower Mesomediterranean	Lower Dry
Jerez		x	Upper Thermomediterranean	Upper Dry
Kalampta	x		Upper Thermomediterranean	Lower Subhumid
Kalfa (Me)	x		Lower Mesomediterranean	Lower Subhumid
La Nucia la marina baixa		x	Upper Thermomediterranean	Lower Dry
Lampedusa	x		Upper Inframediterranean	Lower Semiarid
Larnaka	x		Lower Thermomediterranean	Lower Semiarid
Las Cabezas	x		Upper Thermomediterranean	Lower Dry
Lesina	x		Lower Mesomediterranean	Upper Dry
Linares Ciudad Camino		x	Lower Mesomediterranean	Lower Dry
Linosa	x	x	Lower Thermomediterranean	Lower Semiarid
Lipari	x		Lower Thermomediterranean	Upper Dry
Lopadusa	x	x	Upper Inframediterranean	Lower Semiarid
Los Yesares		x	Upper Thermomediterranean	Lower Semiarid
M. Grasso (Sr),	x		Upper Thermomediterranean	Lower Dry
M. Mela (Ag)	x		Upper Thermomediterranean	Lower Dry
Mallorca Algaida		x	Lower Mesomediterranean	Upper Dry
Marettimo	x		Lower Thermomediterranean	Upper Semiarid
Marmolejo		x	Upper Thermomediterranean	Lower Dry
Mogador (actual Esauria)	x		Lower Thermomediterranean	Upper Semiarid
Monte Kalfa (Me)		x	Lower Mesomediterranean	Lower Subhumid
Morón de la Frontera		x	Upper Thermomediterranean	Upper Dry
Mota del Cuervo		x	Upper Mesomediterranean	Lower Dry
Nador	x		Lower Thermomediterranean	Lower Semiarid
Nafplio	x		Upper Thermomediterranean	Lower Dry
Noto (Sr)	x		Upper Thermomediterranean	Lower Dry
Novelda	x		Upper Thermomediterranean	Upper Semiarid
Oia (Santorini)		x	Lower Mesomediterranean	Lower Dry
Oropesa la plana alta		x	Upper Thermomediterranean	Lower Dry
Oujda		x	Upper Thermomediterranean	Upper Semiarid
Pantano Bruno, Pozzallo (Rg)		x	Lower Thermomediterranean	Lower Dry
Pantano Longarini	x		Lower Thermomediterranean	Lower Dry
Paterna	x		Upper Thermomediterranean	Lower Dry
Pellaro		x	Lower Thermomediterranean	Lower Subhumid
Pellaro	x		Lower Thermomediterranean	Lower Subhumid
Pentidattilo		x	Lower Mesomediterranean	Lower Subhumid
Petra – Olimpa	x		Upper Thermomediterranean	Upper Semiarid
Piana di Catania	x		Upper Thermomediterranean	Lower Dry
Piana di Soluq û ana	x		Lower Thermomediterranean	Lower Semiarid
Porcuna San Pantaleón		x	Lower Mesomediterranean	Lower Dry
Ramath-Gan	x		Upper Inframediterranean	Lower Dry
Riba-Roja camp de túria		x	Upper Thermomediterranean	Lower Dry
Río Anzur		x	Lower Mesomediterranean	Upper Dry
Río Lucena		x	Upper Mesomediterranean	Upper Dry
S. Pedro do Estoril		x	Upper Thermomediterranean	Lower Subhumid
S.Nicola da Crissa, Serre (VV),	x		Upper Thermomediterranean	Lower Subhumid
Sagunto (V)		x	Upper Thermomediterranean	Lower Dry
Sebchet Bu Giarrar	x		Upper Inframediterranean	Lower Semiarid
Tarifa	x		Lower Thermomediterranean	Lower Subhumid
Terme di San Calogero	x		Lower Thermomediterranean	Upper Dry
Termini Imerese	x		Lower Thermomediterranean	Lower Dry
Thyra (Santorini)		x	Lower Mesomediterranean	Lower Dry
Tocra	x		Upper Inframediterranean	Lower Semiarid
Tolmeta	x		Upper Inframediterranean	Lower Semiarid
Tolmeta	x		Upper Inframediterranean	Lower Semiarid
Úbeda		x	Lower Mesomediterranean	Lower Dry
Valle del Guadiato	x		Lower Mesomediterranean	Upper Dry
Varazze		x	Lower Mesotemperate	Lower Subhumid
Vejer	x		Lower Thermomediterranean	Upper Dry
Villaviciosa de Córdoba		x	Lower Mesomediterranean	Upper Dry
Wadi El û bab	x		Upper Inframediterranean	Upper Semiarid
Xabia	x	x	Lower Thermomediterranean	Lower Dry
Xátiva	x	x	Upper Thermomediterranean	Upper Dry
Yávota		x	Lower Mesomediterranean	Lower Dry
**TOTAL**	**71**	**64**		
